# Non‐surgical rhinoplasty through minimal invasive nose thread procedures: Adverse effects and prevention methods

**DOI:** 10.1111/srt.13590

**Published:** 2024-01-26

**Authors:** Soo Yeon Park, Soo‐Bin Kim, Atchima Suwanchinda, Kyu‐Ho Yi

**Affiliations:** ^1^ Made‐Young Plastic Surgery Clinic Seoul South Korea; ^2^ Division in Anatomy and Developmental Biology, Department of Oral Biology Human Identification Research Institute, BK21 FOUR Project, Yonsei University College of Dentistry Seodaemun‐gu Seoul South Korea; ^3^ Department of Dermatology Chulabhorn Hospital, Chulabhorn Royal Academy Bangkok Thailand; ^4^ Department of Dermatology Chulabhorn International Collage of Medicine, Thammasat University Bangkok Thailand; ^5^ Division of Dermatology, Department of Medicine, Faculty of Medicine Ramathibodi Hospital, Mahidol University Bangkok Thailand; ^6^ Maylin Clinic (Apgujeong) Seoul South Korea

**Keywords:** adverse effects, complications, Non‐surgical rhinoplasty, nose thread procedures, prevention methods

## Abstract

**Background:**

This review addresses the intricacies of non‐surgical rhinoplasty, particularly focusing on the utilization of absorbable sutures known as “Volumizing threads” in combination with fillers. The aim is to explore the enhanced precision of nasal contouring offered by these combined procedures compared to sole filler injections.

**Methods:**

Through comprehensive clinical cases, this article scrutinizes the landscape of adverse effects and their prevention strategies associated with minimal invasive nose thread procedures. The discussion emphasizes various complications, including thread protrusion, migration, infections, skin dimpling, and granuloma formation, along with their respective management approaches.

**Results:**

This article delineates cases of complications arising from thread placement, ranging from visibility issues to skin infections and granuloma formation. It highlights instances of thread visibility, oral mucosa protrusion, skin infections, dimpling, and granuloma formation. Additionally, it outlines the corresponding management strategies, accentuating the criticality of early intervention to preclude severe complications in non‐surgical rhinoplasty involving nose threads.

**Conclusion:**

Non‐surgical rhinoplasty, leveraging nose thread procedures, offers heightened precision compared to conventional filler injections. However, the review underscores the importance of recognizing potential risks and promptly addressing complications like thread extrusion, migration, and infections. Understanding these complexities in non‐surgical rhinoplasty aids in informed decision‐making and efficient patient care.

## 1 INTRODUCTION

Recent non‐surgical rhinoplasty procedures involve a combination of absorbable sutures, so called “Volumizing thread” and fillers.^1^ By incorporating threads alongside fillers, it has become possible to achieve more precise and delicate nasal contouring compared to the previous method that solely relied on filler injections. When utilizing threads designed to support and reinforce tissues within the nose, they aid in creating a framework that sustains the nasal bridge and tip. Consequently, this contributes to an extended duration of the procedure's efficacy. Moreover, employing these threads reduces the quantity of filler required, thereby preventing the occurrence of an “Avatar nose” (an overly broad‐looking nose) resulting from excessive filler usage.^2^

Furthermore, it is noteworthy that utilizing threads alone can enhance nose contouring. In addition, due to its shorter procedure time, quicker recovery period, and rising risks linked to fillers, non‐surgical rhinoplasty is steadily gaining traction in South Korea.^3^, ^4^, ^5^

**FIGURE 1:**
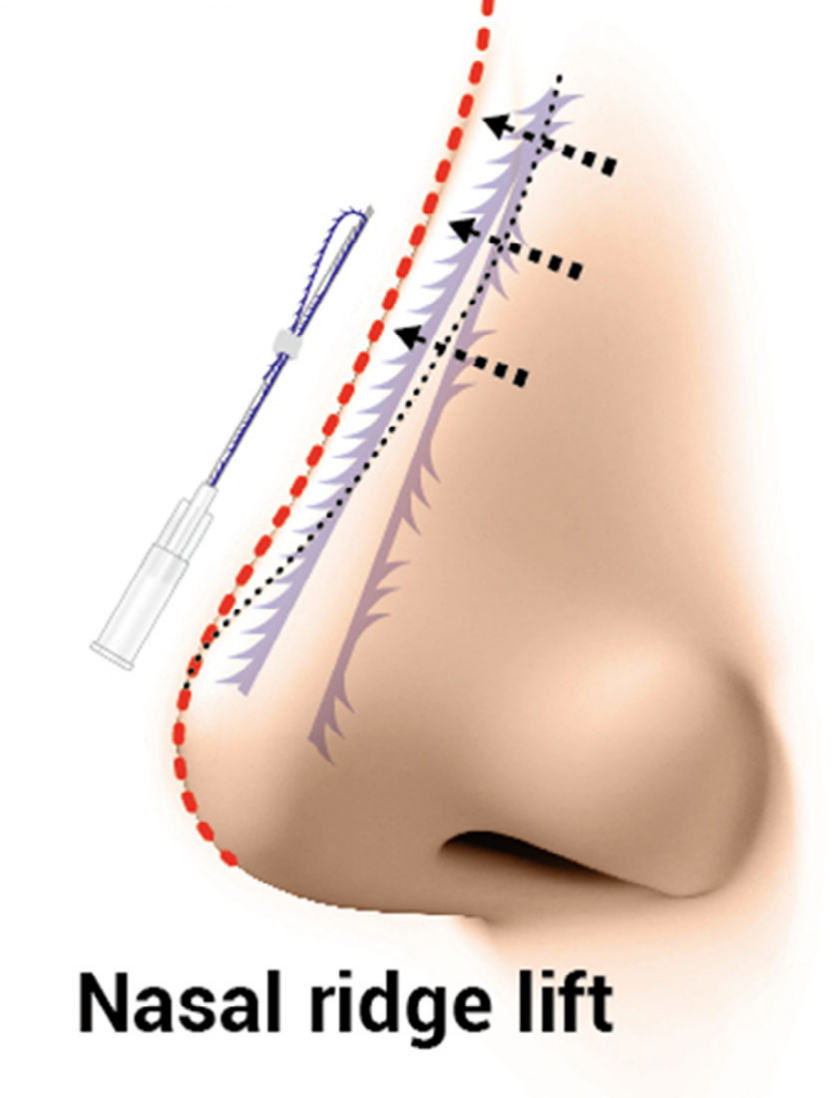
Threads of relatively longer length are predominantly utilized for correcting the nasal bridge. The needle gauge of 21 gauge and needle length of 60 mm (Y‐ko, N‐finders, Inc., South Korea).

**FIGURE 2:**
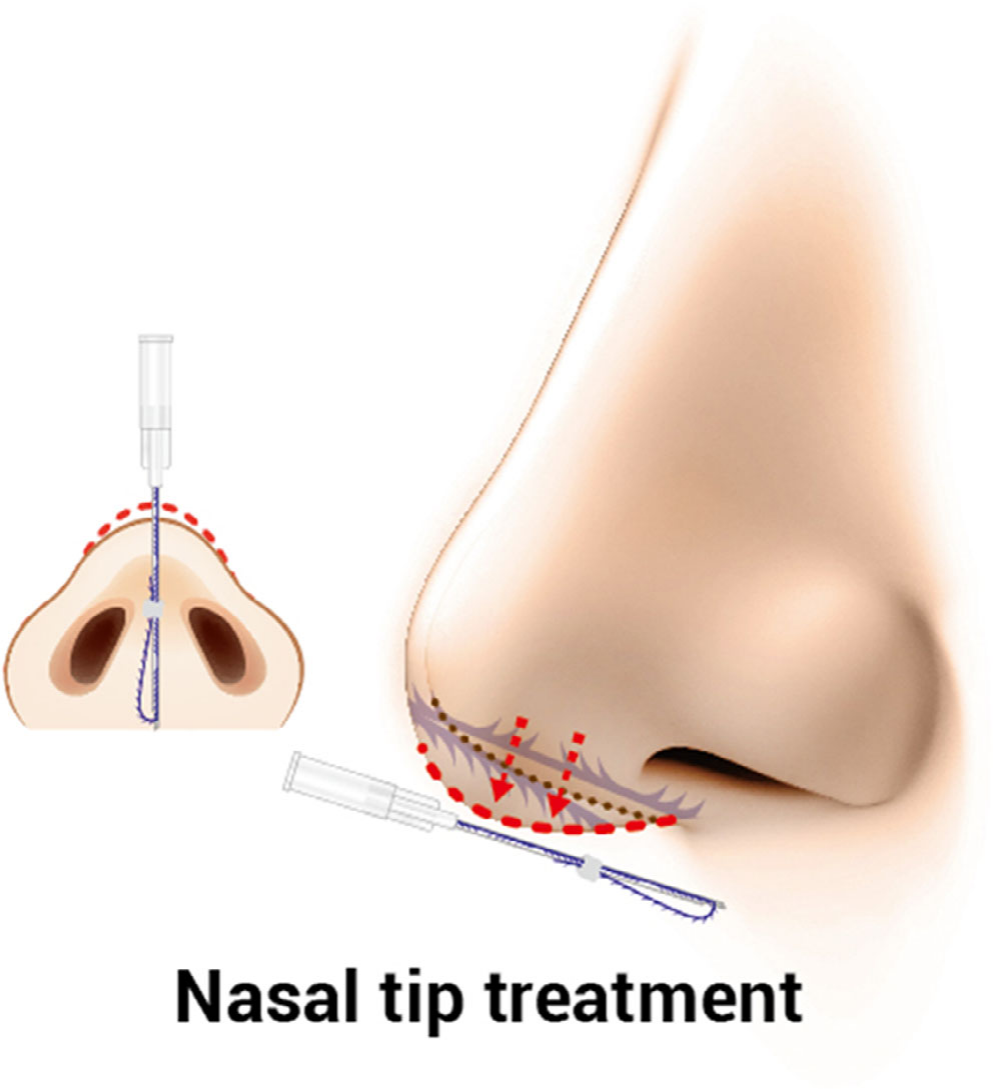
Threads are employed in the nasal tip region to elevate it with 19 gauge needle with length of 40 mm . (Y‐ko, N‐finders, Inc., South Korea).

Typically, practitioners commonly use cog threads equipped with barbs, although they also employ smooth monofilament threads. Threads of relatively longer length are predominantly utilized for correcting the nasal bridge, whereas shorter threads are employed in the nasal tip region to elevate it (Figures 1 and 2). Although complications such as tissue necrosis or blindness can potentially arise due to concurrent use of threads and fillers, these are rare occurrences. However, the authors aim to discuss the causes and solutions regarding post‐procedural side effects following nose thread insertion. While general symptoms like swelling or bruising tend to improve without specific intervention, the authors intend to delve more deeply into addressing uncommon yet severe complications.

## 2 THREAD PROTRUSION, EXTRUSION AND MIGRATION

When performing nasal dorsum thread insertion along the nasal bridge towards the nasal tip, if the threads are shallowly placed and visible enough to be touched or seen through the skin, it may lead to protrusion through the skin if left unattended. If threads are palpable immediately after the procedure, it indicates an improper insertion plane, necessitating immediate removal. In cases involving cog threads, retrograde removal from the entry point may be challenging due to tissue entanglement, leading to inadequate thread removal. In instances where threads are palpable or visible, creating a small incision at the entry point opposite the affected area allows for inspection and complete removal of the thread end using a needle holder.

**FIGURE 3:**
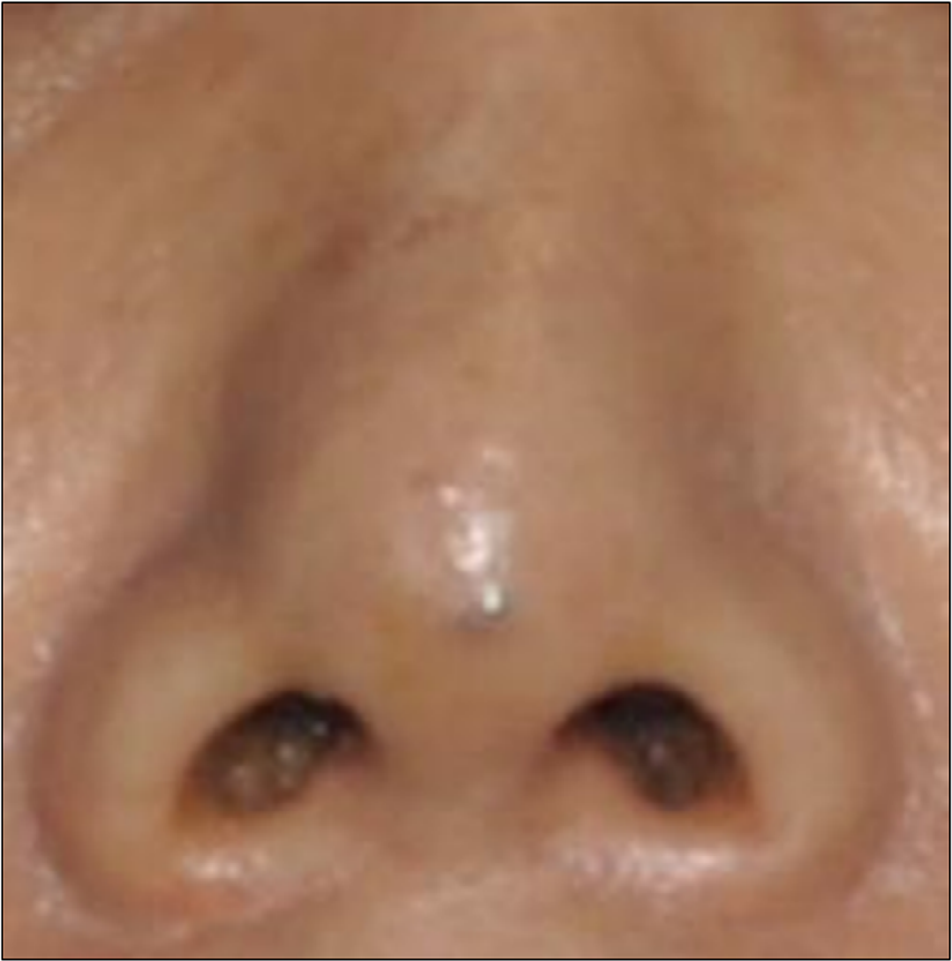
Three weeks after the procedure, the patient presented with persistent pain and visibility of threads at the nasal tip. Using an 18 gauge needle, puncture was performed, followed by retrograde removal of the threads from the entry point."

In some cases, threads may become visible not on the dorsum but at the entry point or nasal tip immediately post‐procedure or over time (Figure 3). Patients may complain of persistent clots rather than visible threads. Despite the inconvenience, it is crucial to ensure patient follow‐up for evaluation. Given the higher likelihood of threads becoming exposed as they dissolve, timely removal remains essential.

**FIGURE 4:**
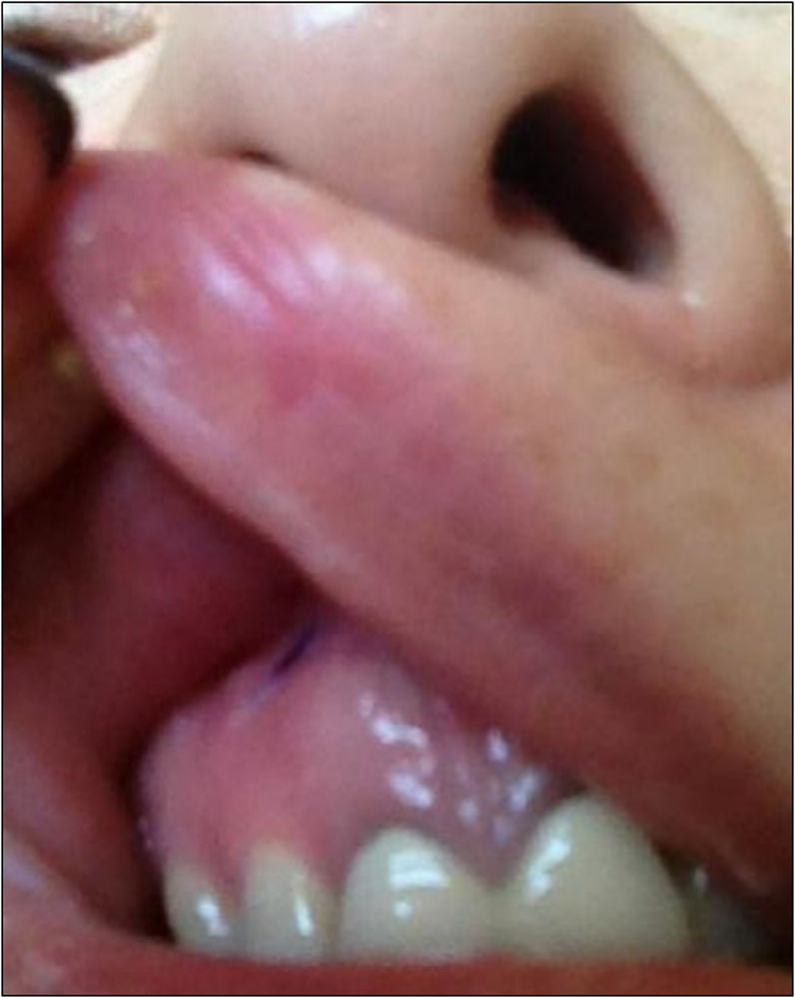
After the procedure, if a cog thread protrudes through the oral mucosa, it can be removed using a needle holder without creating a separate incision.

**FIGURE 5:**
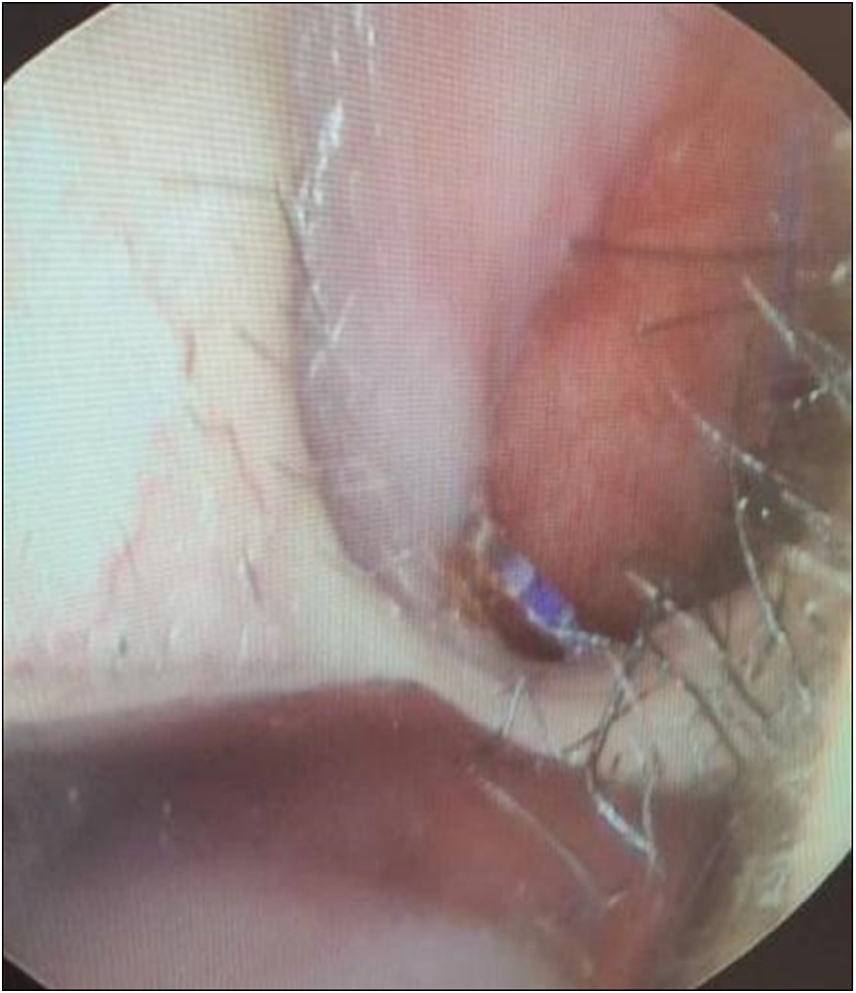
After experiencing persistent discomfort inside the nose post‐procedure, an endoscopic examination revealed a cog thread protruding through the inner lining. The thread end was grasped using a needle holder and completely removed to address the issue.

When performing nasal tip thread insertion along the columella, particularly following the nasolabial angle, there are instances where the thread end may protrude through the oral mucosa (Figure 4). Immediately post‐procedure, it is necessary to examine the inside of the mouth to ensure there is no thread protrusion. If any thread is found to protrude, it can be promptly removed through the oral cavity. Additionally, if a patient complains of persistent discomfort after the procedure, even if no visible thread protrusion is evident on the skin, it might be advisable to examine the inner nasal mucosa using an endoscope or nasal scope (Figure 5). This discomfort might be due to the thread not being positioned in the midline and instead piercing through the inner mucosa, which offers relatively less resistance. In such cases, grasping and directly removing the visible thread usually resolves the issue, and if the mucosa in the wider area is not torn, it typically heals without requiring additional suturing.

In all cases, complete removal of nasal threads is essential as leaving any remnants might necessitate incision later. Actions such as stretching the philtrum immediately after the procedure could induce thread migration, leading to subsequent protrusion through the oral cavity. Similarly, using a needle holder to grasp and remove the thread end usually resolves the issue without complications.

## 3 SKIN INFECTION

**FIGURE 6:**
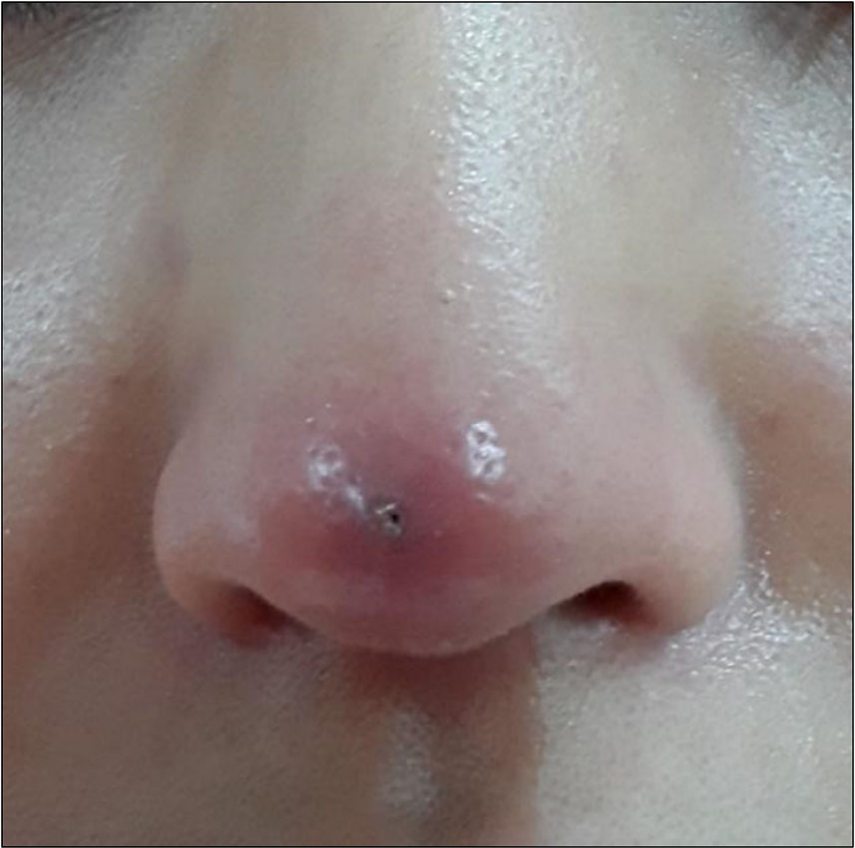
After an infection occurred following a nasal tip procedure using polydioxanone (PDO) cog threads, the condition improved following thread removal and administration of oral antibiotics. There were no lasting effects such as pigmentation.

When aseptic preparation is inadequate during the procedure or if slightly protruding threads are not entirely removed, rare instances of ascending infection can occur (Figure 6). Infections are exceptionally rare when performing polydioxanone (PDO) thread insertion alone. However, in cases where previous implant insertion or open rhinoplasty involving foreign bodies such as silicone exists, PDO threads, known for their high resorbability, might undergo catalysis and concurrently act as a potential source of infection. Therefore, a cautious decision must be made regarding the necessity of nasal thread procedures in patients with a relevant medical history. Specifically, as the nasal tip area has relatively lower vascular circulation, it is more susceptible to infection, necessitating increased vigilance.

Mild infections without an open wound usually respond well to conservative treatment. However, it is imperative to remove the threads and initiate antibiotic therapy.^6^ If a previous implant exists, complete surgical removal might be necessary. In our clinic, for patients without a history of specific surgical procedures, we prescribe Cephalosporin antibiotics for approximately 3–7 days. In cases with a history of previous implant insertion or severe involvement of nasal mucosa in the wound, there might be a possibility of Methicillin‐Resistant Coagulase‐Negative Staphylococci (MRCN) or Methicillin‐Resistant Staphylococcus Aureus (MRSA). Therefore, we recommend broad‐spectrum antibiotics such as clindamycin, trimethoprim‐sulfamethoxazole (cotrimoxazole), doxycycline, minocycline, linezolid, considering the likelihood of these bacteria.^7^ Prescription of antibiotics is further guided by the results of wound culture and antibiotic susceptibility testing.

## 4 SKIN DIMPLING

**FIGURE 7:**
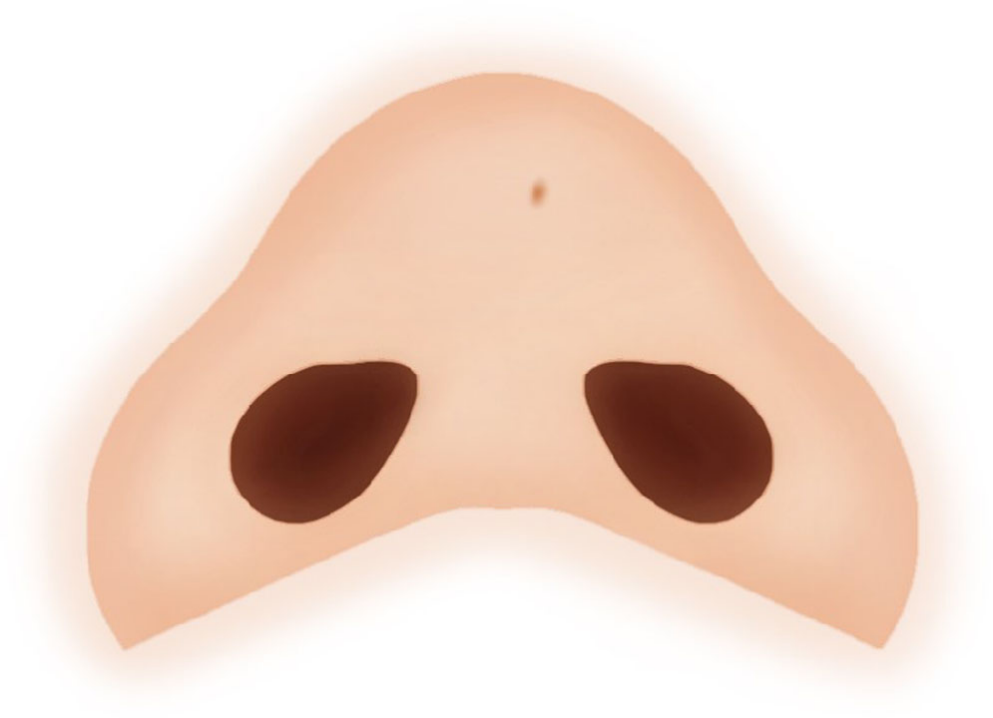
After undergoing rhinoplasty using polydioxanone (PDO) monothreads, the patient experienced persistent erythema and swelling for about a month, followed by the development of a depressed scar at the nasal tip. Treatment involved laser therapy and fat grafting to address the issue.

When the insertion point of PDO threads penetrates the dermis, occasionally resulting in an immediate dimple, this can be rare but manageable through procedures like subcision.^8^ However, in cases where inflammation persists, it may lead to delayed dimpling due to contracture (Figure 7).^9^ Patients with a history of prior surgeries are at a higher risk of contracture. While inducing tissue regeneration using fractional laser or tissue‐restorative biomaterials is possible, reversal of contracture is challenging, emphasizing the importance of preventive measures. Injecting micronized fat grafts into scars can offer assistance. Contractures often occur as a secondary complication following issues like infection; hence, cog thread removal should be considered if persistent erythema and swelling are present initially. Removal of monothreads might necessitate surgical intervention due to the difficulty in extraction.

## 5 GRANULOMA FORMATION

**FIGURE 8:**
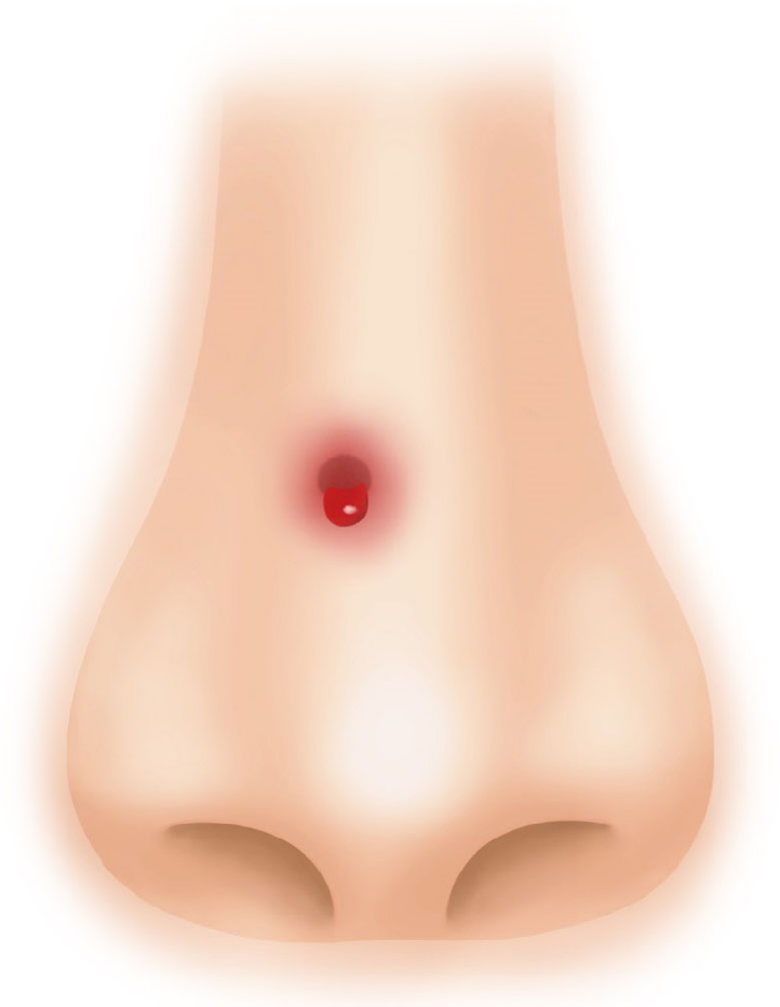
Patient with a history of open rhinoplasty using acellular dermal matrix around 20 years ago, underwent additional non‐surgical rhinoplasty using polydioxanone (PDO) monothreads approximately 5 months ago, subsequently developed a supratip‐lobule.

In rare instances, patients with a history of previous nasal surgeries who undergo additional non‐surgical rhinoplasty using threads may exhibit granuloma formation (Figure 8).^9^ PDO threads, due to their high absorption rate and simultaneous promotion of collagen synthesis alongside inducing foreign body reactions, can lead to surrounding inflammation, potentially creating an open wound.^10^ In such cases, the development of foreign body granulomas accompanied by a fibrous capsule within the wound area is likely, necessitating the possibility of a direct incision through the nasal dorsum.^11^

## 6 DISCUSSION

When selecting appropriate candidates for thread rhinoplasty, more effective procedures can be achieved compared to using fillers alone. However, early complications such as thread extrusion or protrusion during the procedure need immediate and proactive correction to prevent severe complications. Early and proactive intervention is crucial to avoid progressing into severe complications, and surgical intervention may be necessary if required. Based on the author's experience, undergoing nose contouring procedures with threads at intervals of approximately 6 months, repeated 2–3 times, can be performed without significant issues. However, in cases where patients desire more repeated procedures, it might lead to the formation of fibrotic tissue, making it challenging to achieve the desired outcome. In such instances, consideration of open rhinoplasty becomes necessary. During surgery, tissue scarring may complicate dissection, making the procedure more challenging, hence requiring careful explanation and consideration with the patient before proceeding.

Non‐surgical rhinoplasty, particularly involving minimal invasive nose thread procedures, has emerged as a viable alternative to traditional surgical approaches, offering precise nasal contouring and augmentation. Incorporating absorbable sutures alongside fillers has revolutionized the field, allowing for delicate enhancements and prolonged efficacy compared to fillers alone.

However, this comprehensive review underscores the significance of understanding and managing potential complications arising from these procedures. Adverse effects such as thread extrusion, migration, infections, dimpling, and granuloma formation, albeit uncommon, necessitate prompt intervention to prevent severe complications.

Recognizing the nuances in managing these complications is crucial for practitioners performing non‐surgical rhinoplasty. Timely and proactive measures, including prompt removal of threads and appropriate antibiotic therapy, are imperative to mitigate complications and ensure favorable outcomes.

Moreover, patient selection plays a pivotal role in mitigating risks associated with these procedures. While repeated interventions at intervals show promising results, excessive treatments may lead to challenges like fibrotic tissue formation, prompting consideration of alternative approaches such as open rhinoplasty.

In conclusion, non‐surgical rhinoplasty through minimal invasive nose thread procedures offers significant aesthetic benefits but demands comprehensive knowledge, vigilance, and a meticulous approach in handling potential complications to ensure patient safety and optimal outcomes. Continued research and refined techniques will further advance the safety and efficacy of these procedures in the realm of esthetic rhinoplasty.

## CONFLICT OF INTEREST STATEMENT

I acknowledge that I have considered the conflict of interest statement included in the “Author Guidelines.” I hereby certify that, to the best of my knowledge, that no aspect of my current personal or professional situation might reasonably be expected to significantly affect my views on the subject I am presenting.

## Data Availability

The data that support the findings of this study are available from the corresponding author upon reasonable request.
